# Correlations between the prescribing patterns of psychotropic medications and socio-economic factors during the COVID-19 pandemic: A cross-sectional Swedish registry study

**DOI:** 10.1371/journal.pone.0330081

**Published:** 2025-09-17

**Authors:** Dainty Ei, Gunnar Brådvik, Peter Lindgren, Paul Barach, Tomasz Bochenek

**Affiliations:** 1 Department of Nutrition and Drug Research, Institute of Public Health, Faculty of Health Sciences, Jagiellonian University Medical College, Krakow, Poland; 2 Impact Assessment Unit, Office for Service Transformation, Singapore Health Services, Singapore; 3 The Swedish Institute for Health Economics, Lund, Sweden; 4 Department of Learning, Informatics, Management and Ethics, Karolinska Institutet, Stockholm, Sweden; 5 Thomas Jefferson University, Philadelphia, Pennsylvania, United States of America; 6 University of North Carolina, North Carolina, United States of America; University of Botswana, BOTSWANA

## Abstract

**Background:**

The COVID-19 pandemic has had a profoundly negative impact on all societal sectors, public health systems, and state economies. The pandemic led to high levels of stress, anxiety, depression, insomnia, and substance abuse, while the impact on changes in psychotropic medication prescribing was complex. Despite less restrictive measures in the first stage of the pandemic, Sweden experienced significant mental health consequences and changes in psychotropic medication prescribing.

**Aim:**

This study aimed to characterize the different psychotropic medication prescribing patterns (antidepressants, anxiolytics, hypnotics and sedatives, and drugs used in addictive disorders: nicotine, alcohol, and opioid dependence) at regional levels and investigate the correlations of disease spread and socio-economic factors with the Swedish regional prescribing patterns during the COVID-19 pandemic.

**Methods:**

We employed an observational and retrospective design, incorporating time-series analysis, spatial visualization, and regression analysis.

**Results:**

The prescribing of anxiolytics and drugs used in addictive disorders decreased during the pandemic, with the most striking reductions seen in medications used for nicotine dependence. Considerable regional variations were observed across Sweden during the pandemic years, with antidepressant prescribing increasing slightly, and hypnotics and sedatives showing a relatively stable trend. None of the four key variables of disease spread and socio-economic factors showed a statistically significant correlation with the changes in the prescribing of drugs used for nicotine dependence.

**Conclusions:**

Our study demonstrated differentiated changes in psychotropic medication prescribing patterns during the COVID-19 pandemic in Sweden. We found a pandemic effect on nicotine dependence drug prescribing, which the key variables could not explain. Targeted mental health interventions and specific regional health policies should be developed to reduce disparities and address regional variations during future health emergencies.

## Introduction

Decision-makers in the healthcare systems should not ignore the lessons of the COVID-19 pandemic, which negatively impacted all societies. From the economy to education, social interactions to personal relationships, no domain was left unaffected by the pandemic. The growing mental health crisis is regarded as one of the most significant consequences of the COVID-19 pandemic. The sudden disruptions in daily life, prolonged periods of social isolation, fear of unknown futures and infections, loss of loved ones, and economic uncertainties have all contributed to high silent mental health challenges, including anxiety, stress, depression, and substance abuse [1,2].

The prevalence of adverse mental health outcomes increased significantly during the pandemic, especially among vulnerable groups such as healthcare workers, essential workers, young adults, the elderly, and individuals with pre-existing mental health conditions [3]. A study of eight countries from three continents revealed that the primary symptoms resulting from the pandemic included anxiety (42%), depression (43%), and suboptimal social connectedness and self-confidence ratings (38%) [4]. The rates of insomnia were also high, with 37% in the first wave of COVID-19, especially in at-risk populations in Western countries [5]. A systematic review and meta-analysis found that 33% of adults experienced anxiety and 28% depression during the pandemic. Another systematic review evidenced the high rates of psychological distress, mainly stress (8–82%), depression (15–48%), anxiety (6–51%), and post-traumatic stress disorder (7–54%) among a general population sampled from eight countries, and was more prominent in women, adults aged less than 40 years, individuals with chronic or psychiatric illnesses, unemployed persons, and students [2].

In Sweden, a population-based survey conducted in April and May 2020 found significantly increased rates of depression (30%), anxiety (24.2%), and insomnia (38%) among adults [6]. A cross-sectional study found clinically significant depression and anxiety in the general population, with the prevalence of 22.2% and 28.3%, respectively, in the early stage of the pandemic [7]. The symptoms were more prominent in the younger population, unemployed individuals, and populations with pre-existing mental health problems and financial instability. The higher stress levels among children and youth in Sweden were attributed to increased sedentary behavior and a decrease in physical activity during the pandemic [8]. During the first COVID-19 wave, an increase in generalized anxiety and depression symptoms, resulting from physical inactivity and negative social and lifestyle changes, was also seen in the Swedish working population [9]. Clear consequences of the pandemic were seen with higher gambling rates, increased alcohol consumption, and psychological distress among Swedes [10]. A study comparing health-related quality of life (HRQoL) levels in the Swedish population reported significant reductions in HRQoL during the pandemic compared to the pre-pandemic period, particularly in working-age adults [11].

However, studies based on healthcare utilization and insurance data have shown mixed findings, with some indicating declines in diagnoses and treatment, as illustrated in the following examples. In the Scania Region of Sweden, no significant changes were seen in prescribing trends for most psychotropics following the pandemic outbreak [12]. A substantial decline in seeking treatment for emergency psychiatry, especially anxiety and mood disorders, was reported during the pandemic in Sweden. This selective reduction in emergency visits may have been related to concerns about COVID-19 exposure in emergency care settings [13]. Similarly, a nationally representative survey in the United States (US) found that while mental distress increased during the pandemic, treatment-seeking did not rise proportionately [14]. In Germany, the prescribing of psychotropics for newly diagnosed patients during the pandemic was lower than in previously diagnosed patients [15]. Other studies captured the decreasing trends of psychotropic medication use in Norway and Sweden and the increasing trends in France and Denmark [16–18]. The incidence of depression and anxiety diagnoses, antidepressant prescribing, and self-harm declined significantly in the UK after March 2020, only returning to pre-pandemic levels by September 2020 [19]. Internationally, despite the rising rates of mental health issues during the pandemic, some studies reported increases in the utilization of psychotropic drugs [20–24], whereas others found no significant changes or even declines [12–15,19]. One study in Sweden found an increase in weekly prescription drug dispensing and sales of over-the-counter (OTC) drugs during the early pandemic [25].

This study aimed to characterize the different patterns of psychotropic medication prescribing (defined as antidepressants, anxiolytics, hypnotics and sedatives, and drugs used in addictive/substance use/ disorders: nicotine, alcohol, and opioid dependence) at regional levels and investigate the correlations of disease spread and socio-economic factors with these prescribing patterns at regional levels in Sweden during the COVID-19 pandemic. The research objectives include: i) examining the prescribing patterns of select psychotropic medications in Sweden during the COVID-19 pandemic using public data; ii) analysing temporal trends and regional variations in prescribing patterns for each drug category by identifying all significant deviations from pre-pandemic trends; iii) exploring the correlations between the prescribing patterns of psychotropics and the disease spread and socio-economic factors in different regions of Sweden during the pandemic. The null hypothesis in this study was that no significant changes occurred in the prescribing of selected psychotropic medications during the pandemic compared to the pre-pandemic period, and there were no geographical differences in this regard.

The study results could guide addressing the mental health consequences of large-scale health emergencies that may arise in the future.

## Materials and methods

We employed an observational and retrospective design, including a time-series analysis, spatial visualization, and regression analysis.

### Study population and sampling

The study population comprises all Swedish residents living in all 21 regions, each with its regional government responsible for public healthcare and public transportation [26,27]. All age groups and genders were included, and no exclusion criteria were applied. While the overall number of Swedish residents was 10,378,483 in 2020 (with 50.32% males), it increased to 10,443,100 in 2021 (males – 50.33%), and further to 10,514,719 in 2022 (males – 50.35%). The demographic distribution of the Swedish population by 21 regions for the years 2020, 2021, and 2022 is available in the S1 Data [28]. A detailed description of Sweden’s geographical administrative division [29] can be found in the S2 Table. The data was included from individuals in Sweden who had been dispensed the selected psychotropic drugs from 2014 to 2022 to analyse changes before and during the COVID-19 pandemic. All data were collected at the regional level in all 21 Swedish counties [30].

### Data sources – general information

The source data used in this study were obtained from publicly available Swedish Government databases, accessed on June 14, 2024 (medicines used and socioeconomic factors), and August 2, 2024 (additional information for regression analysis).

### Data sources – measures of psychotropic medications (dependent variable)

The drug prescribing data were obtained from the National Prescribed Drug Register, maintained by the National Board of Health and Welfare (Socialstyrelsen) [31,32]. Hospital prescribing and OTC medications were not included in this database. The specific drug classes, according to the Anatomical Therapeutic Chemical (ATC) Classification system, included in this study are: N05B ‘Anxiolytics’; N05C ‘Hypnotics and sedatives’; N06A ‘Antidepressants’; N07B ‘Drugs used in addictive disorders’; N07BA ‘Drugs used in nicotine dependence’; N07BB ‘Drugs used in alcohol dependence’; N07BC ‘Drugs used in opioid dependence’.

To quantify drug prescribing patterns, ‘the number of patients per 100,000 inhabitants’ was used as a proxy measure for ‘the mental health drug prescribing rates’ for each ATC group at the county level for each year. Two periods were selected: the pre-COVID-19 (2015–2019) and the COVID-19 (2020–2022); see the S3 Text for detailed information. The range of years from 2014 to 2019 was included for the baseline trend, and 2020–2022 for pandemic year changes. Data on post-pandemic years were excluded.

### Data sources – measures of socio-economic factors (independent variables)

All data on variables presented below were collected from the National Board of Health and Welfare [30]. The data on COVID-19 patients in hospitals was chosen as a proxy measure for the disease’s spread and labelled as ‘the number of COVID-19 patients in hospital per 1,000 population’ [30]. During the pandemic, it covered all COVID-19 patients in inpatient care, including intensive care units (ICU). The mean income and the post-school education levels were chosen to measure socio-economic factors. The data for the ‘mean income’ variable, presented in Swedish Krona (SEK) in thousands, was obtained from the Household Finances database of Statistics Sweden (Statistiska centralbyrån, SCB) [33]. The average yearly after-tax income (for both genders, aged 20 and older) for each year from 2020−2022, by region, was downloaded. The data for ‘the proportion of the population with post-school education’ variable was obtained from the Education and Research Database of Statistics Sweden [34], covering both genders, from 16 to 74 years old, who completed any post-secondary education. The distance from the capital of each county to Copenhagen, Denmark, was included as one covariate because personal trans-border importing of alcoholic beverages and tobacco products is quite popular in Sweden [35]. This is due to the Swedish monopoly system in alcohol retailing (Systembolaget) and high taxes on these products compared to neighbouring countries [36,37]. The primary sources of purchase remain countries south of Sweden, and the most convenient connection is often via Denmark. The variable, coded as ‘the distance to Copenhagen in kilometres (km)’, was chosen to investigate the association between cross-border purchasing and changes in drug prescribing with regional variation. The S4 Text provides a detailed explanation of the socio-economic data measures, while the source data for analyses performed in this study can be found in the S5 Data. From among the available data, any factors that were not reflective of changes in psychotropic medicine prescribing, according to the study’s aim and objectives (e.g., temperature or percentage of the population testing positive for COVID-19), were excluded.

### Statistical analysis of data

We measured the drug prescribing rates using time-series plots and map plots for each drug group. The different trends of the actual and expected numbers of patients prescribed the chosen drugs during the COVID-19 pandemic were described using time-series plots. The expected number of patients for 2020, 2021, and 2022 was calculated based on the average patient growth rates from 2015 to 2019. Moreover, the percentage differences between actual and expected patient numbers from 2020 to 2022 across the 21 regions were described using spatial visualization, with map plots to examine regional variation.

The relationships between drug prescribing patterns and four independent variables were presented using scatter plots for 2020, 2021, and 2022. The N07BA ‘Drugs used in nicotine dependence’ was chosen to test the correlation, as the analysis had shown a reduction in the consumption of this type of medication throughout the pandemic. For the scatter plots, the ‘percentage difference in the actual number of patients and the expected number of patients’ (previously presented) has been tested against four key variables.

A panel regression model was applied after examining the scatter plots to investigate correlations across the entire period from 2020 to 2022. Using a panel regression, the unobserved heterogeneity was controlled, and individual effects and time effects were captured to explain the relationships between the variables across different regions that change over time. Additionally, for each year during the pandemic, a multiple linear regression model was used to examine the correlations between the primary dependent variable and the three key independent variables. The fourth independent variable (distance to Copenhagen in km) was excluded from the fixed effect model due to its time invariance. All models were weighted by each county’s population (i.e., the number of inhabitants).

To mitigate the selection bias regarding patients using psychotropic medications, all age and gender groups from all 21 regions of Sweden were included. The information bias was mitigated by using data from standardized national registries. The measurement bias was mitigated by using the same measurement approach (‘the number of patients per 100,000 inhabitants’ to quantify drug prescribing patterns) across all regions and periods. The data were weighted by population in the regression analysis to mitigate the regional bias.

We used the statistical computing R software version 4.4.0 (2024-04-24) [38]. The maps were created using swemaps2 in R [39].

### Ethical considerations

The authors had no access to information that could identify any individuals during or after data collection. The study received ethics approval from the University Research Ethics Committee of the University of Sheffield (registration number 220198354, reference number 058312).

## Results

**Graph 1:**
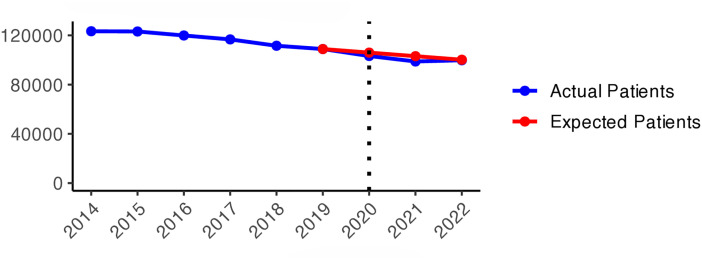
Anxiolytic (N05B) prescribing changes from 2014 to 2022. Actual vs expected patients per 100,000 inhabitants, both genders (source: Socialstyrelsen, 2024).

**Fig 1:**
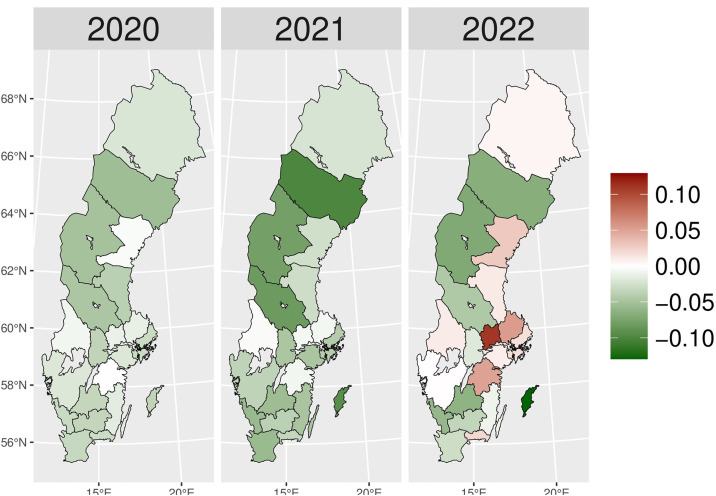
Regional differences in percentage changes of N05B prescribing rates during the pandemic. Actual vs expected patients, both genders. Expected patient data based on the 2015–2019 average growth rate (source: Socialstyrelsen, 2024).

We present the yearly changes in the intensity of prescribing selected medicines expressed as the number of patients per 100,000 inhabitants. We observed a steady decrease in the number of patients prescribed anxiolytics (N05B) from 2014 to 2019 (Graph 1). From 2020 onwards, the actual patient numbers fell below the expected values based on the predicted growth rates from 2015 to 2019. This gap widens slightly in 2020 and 2021, but the values get closer in 2022, suggesting a lower use of anxiolytics during the COVID-19 pandemic than pre-pandemic projections. Fig 1 illustrates the regional variations between actual and expected values of anxiolytic prescribing. In 2020, the most significant negative values (reductions in actual values) were observed in Västerbotten, Dalarna, and Jämtland counties, becoming more pronounced in 2021. However, by 2022, the values had become closer to what was expected in some parts of South and East Sweden, especially in Västmanland County.

**Graph 2:**
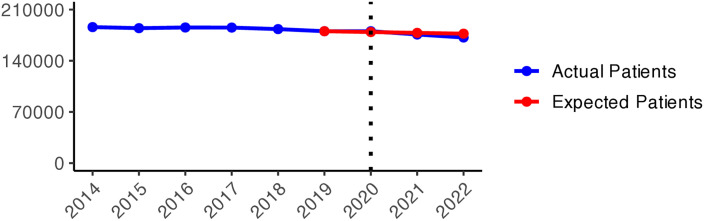
Hypnotic and sedative (N05C) prescribing changes from 2014 to 2022. Actual vs expected patients per 100,000 inhabitants, both genders (source: Socialstyrelsen, 2024).

**Fig 2:**
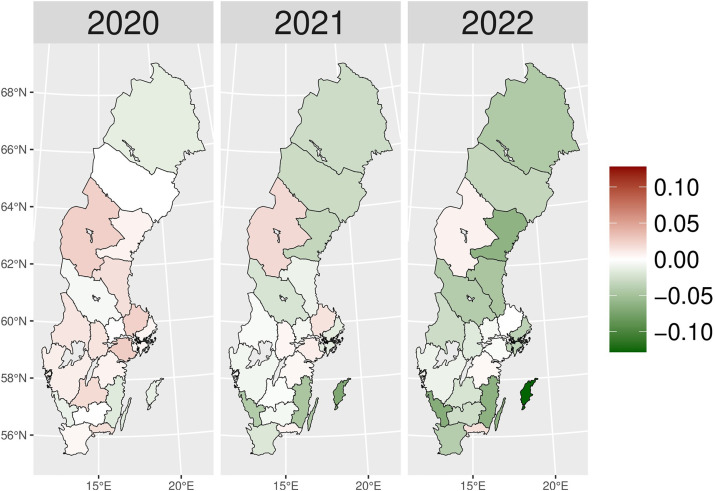
Regional differences in percentage changes of N05C prescribing rates during the pandemic. Actual vs expected patients, both genders. Expected patient data based on the 2015–2019 average growth rate (source: Socialstyrelsen, 2024).

The number of patients who received hypnotics’ and sedatives’ prescriptions (N05C) remained relatively stable over the observed period (approximately 180,000–170,000 patients), with a slight decrease during the pandemic years (Graph 2). Although the number of patients prescribed N05C was slightly higher in 2020 than expected, the values fell below schedule in 2021 and 2022. During the early pandemic period (Fig 2), higher numbers of actual patients compared to expected ones (red areas) were observed in most areas of Sweden. In 2021 and 2022, shifts towards negative values (green areas) were observed in all of Sweden, except in Jämtland and Blekinge counties, indicating fewer prescriptions of hypnotics and sedatives during the later pandemic period.

**Graph 3:**
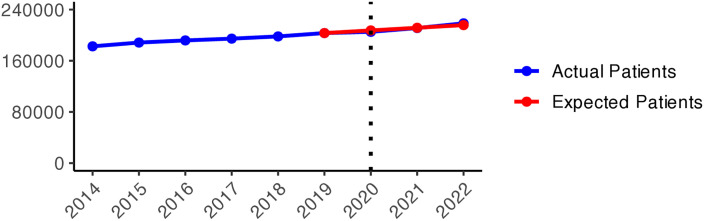
Antidepressant (N06A) prescribing changes from 2014 to 2022. Actual vs expected patients per 100,000 inhabitants, both genders (source: Socialstyrelsen, 2024).

**Fig 3:**
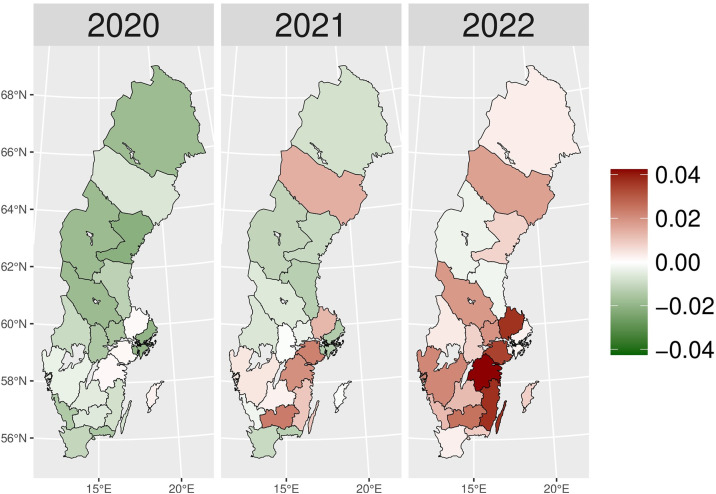
Regional differences in percentage changes of N06A prescribing rates during the pandemic. Actual vs expected patients, both genders. Expected patient data based on the 2015–2019 average growth rate (source: Socialstyrelsen, 2024).

Graph 3 illustrates an increasing trend in prescribing antidepressants (N06A) over the given period. However, a slight decline in the actual values can be observed at the onset of the pandemic, which conflicts with what might have been anticipated. Despite that, the values continued to increase, reaching the expected values in 2021 and slightly exceeding the expected numbers in 2022. While the overall use of antidepressants was somewhat lower than expected at the start of the pandemic in Sweden, the actual values exceeded the expected ones in Västerbotten and East Sweden in 2021. Apart from Gävleborgs and Jämtland, positive changes were experienced in all of Sweden in 2022 (Fig 3).

**Graph 4:**
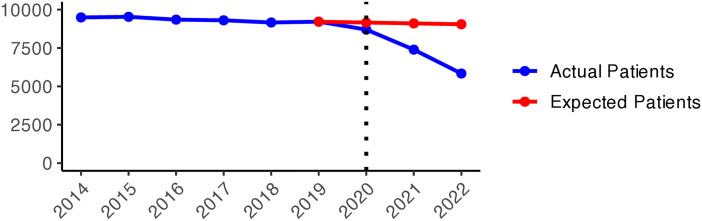
Drugs used in addictive disorders (N07B) prescribing changes from 2014 to 2022. Actual vs expected patients per 100,000 inhabitants, both genders (source: Socialstyrelsen, 2024).

**Fig 4:**
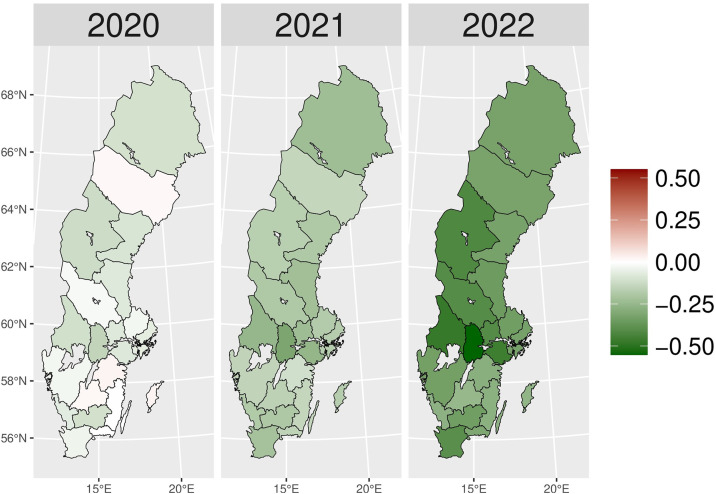
Regional differences in N07B drug prescribing rate percentage changes during the pandemic. Actual vs expected patients, both genders. Expected patient data based on the 2015–2019 average growth rate (source: Socialstyrelsen, 2024).

The utilization of all drugs used in the treatment of substance use disorders (nicotine, alcohol, and opioids – N07B) decreased dramatically during the pandemic period as compared to the relatively constant pre-pandemic prescribing rates (Graph 4). The gaps between actual and expected values widened during the pandemic, reaching a peak in 2022. Although we observed lower actual than expected drug prescribing patterns for addictive disorders in most regions in 2020, Västernorrlands and Kalmar counties showed the opposite results (Fig 4). However, during the later pandemic period, the entire country experienced a significant uniform reduction in drug prescribing compared to predicted values.

**Graph 5:**
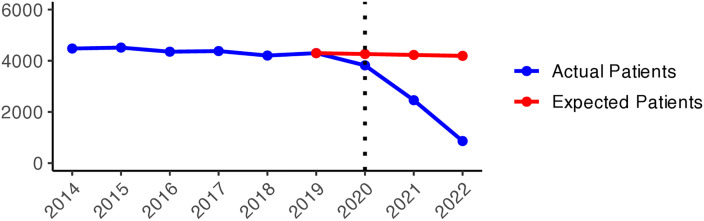
Drugs used in nicotine dependence (N07BA) prescribing changes from 2014 to 2022. Actual vs expected patients per 100,000 inhabitants, both genders (source: Socialstyrelsen, 2024).

**Fig 5:**
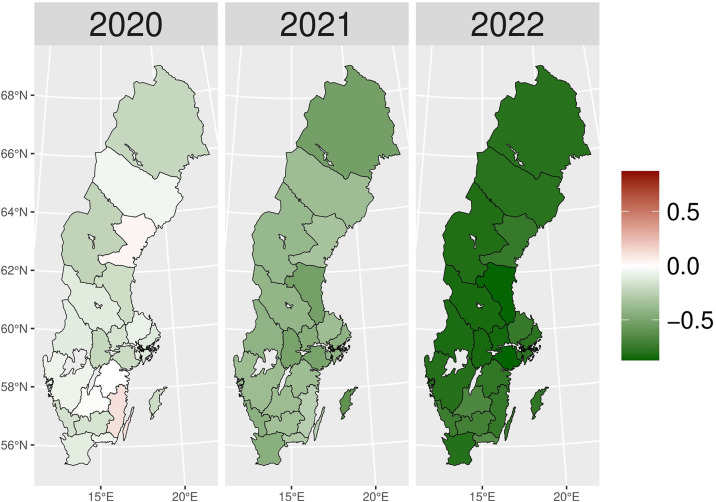
Regional differences in percentage changes of N07BA drug prescribing rates during the pandemic. Actual vs expected patients, both genders. Expected patient data based on the 2015–2019 average growth rate (source: Socialstyrelsen, 2024).

Graph 5 shows a relatively stable trend in nicotine dependence drugs (N07BA) prescribing up to 2019. From 2020 onwards, there was a sharp decline in actual values compared to expectations. The gap widened dramatically over time, and in 2022, the exact value dropped to five times lower than expected. The regional variation of N07BA drugs is similar to that of N07B. In Fig 5, Västernorrlands and Kalmar counties experienced a higher actual patient number than expected in 2020. However, the nationwide decrease in nicotine dependence drug use was seen in the late pandemic, with a more prominent reduction in 2022.

**Graph 6:**
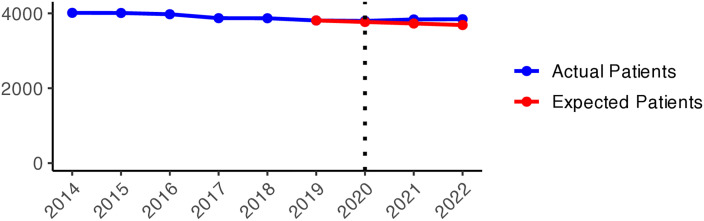
Drugs used in alcohol dependence (N07BB) prescribing changes from 2014 to 2022 (both genders). Actual vs expected patients per 100,000 inhabitants, both genders (source: Socialstyrelsen, 2024).

**Graph 7:**
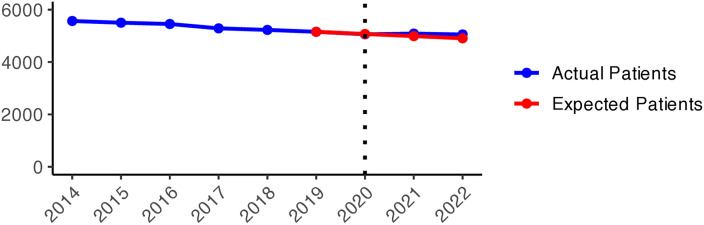
Drugs used in alcohol dependence (N07BB) prescribing changes from 2014 to 2022 (males). Actual vs expected patients per 100,000 inhabitants, males (source: Socialstyrelsen, 2024).

**Graph 8:**
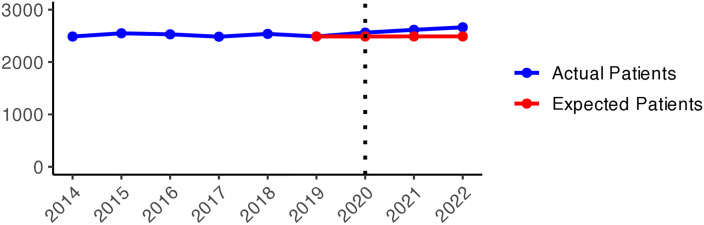
Drugs used in alcohol dependence (N07BB) prescribing changes from 2014 to 2022 (females). Actual vs expected patients per 100,000 inhabitants, females (source: Socialstyrelsen, 2024).

Among the drug classes studied, the N07BB subclass (drugs used in alcohol dependence) shows interesting gender-specific variations in prescribing patterns. Consequently, the descriptive analysis for this subclass includes separate data for males and females, in addition to the overall population data. Graph 6 illustrates the prescribing trend of N07BB drugs for both genders from 2014 to 2022. Although the trend showed a gradual decrease over time, the prescribing increased from 2020 and reached its 2014 value at the end of the pandemic. The male trend (Graph 7) is similar to the overall trend, exhibiting a more pronounced decrease before the pandemic. The male population also showed an increase in the actual prescribing rate, with a narrower gap during the pandemic. In contrast, the female population experienced a broader gap with a substantial increase in actual alcohol dependence drug prescribing throughout the COVID-19 pandemic period, exceeding that of pre-pandemic values (Graph 8). However, the overall value of males was double that of females throughout the period.

**Fig 6:**
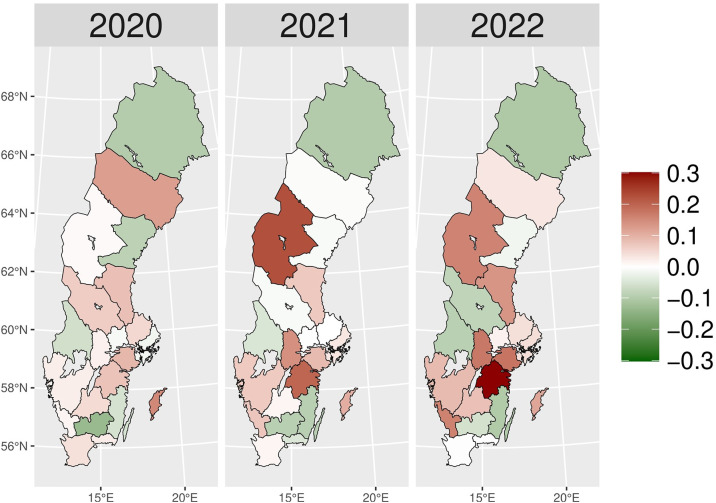
Regional differences in percentage changes of N07BB drug prescribing rates during the pandemic (both genders). Actual vs expected patients, both genders. Expected patient data based on the 2015–2019 average growth rate (source: Socialstyrelsen, 2024).

**Fig 7:**
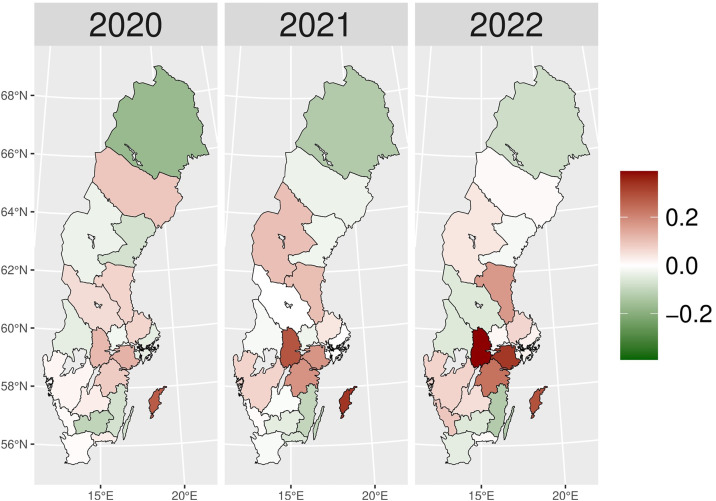
Regional differences in percentage changes of N07BB drug prescribing rates during the pandemic for males. Actual vs expected patients, males. Expected patient data based on the 2015–2019 average growth rate (source: Socialstyrelsen, 2024).

The regional variation for N07BB was relatively heterogeneous for each year during the pandemic. The values for Jämtland County fluctuated, achieving the highest level in 2021. In 2022, Östergötlands County showed the highest actual value, which increased continuously over the pandemic (Fig 6). In Fig 7, the regional difference for alcohol dependence drug prescribing in males shows a pattern that is similar to the one detected for both genders – some counties with growing and some with declining numbers over the period.

**Fig 8:**
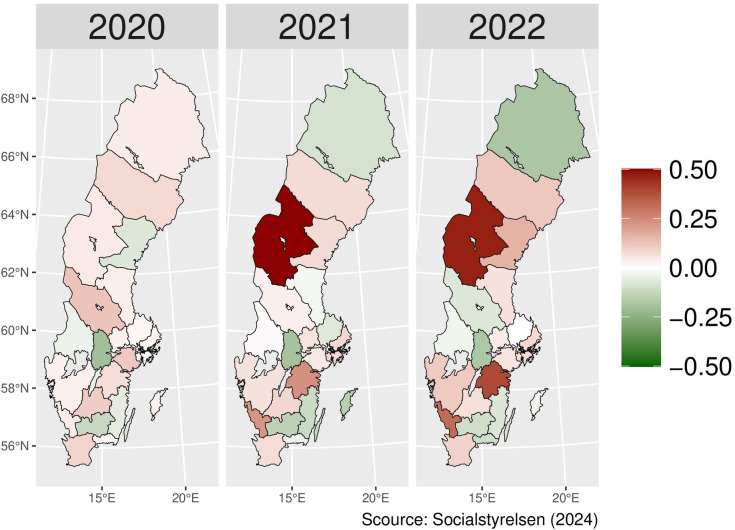
Regional differences in percentage changes of N07BB drug prescribing rates during the pandemic for females. Actual vs expected patients, females. Expected patient data based on the 2015–2019 average growth rate (source: Socialstyrelsen, 2024).

In Fig 8, female regional variation is distinct from the overall pattern, and prescribing rates higher than expected over the period were observed in almost every county in Sweden, except for Norrbotten and Jönköping. However, Dalarna, Gotland, and Östergötlands counties remained the highest for both males and females in 2022.

**Graph 9:**
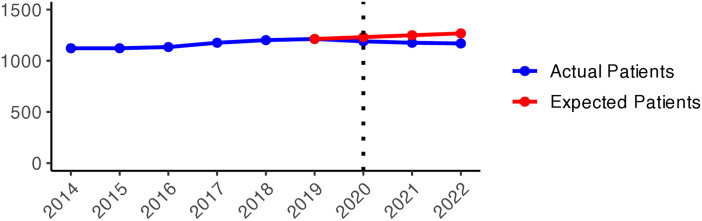
Drugs used in opioid dependence (N07BC) prescribing changes from 2014 to 2022. Actual vs expected patients per 100,000 inhabitants, both genders (source: Socialstyrelsen, 2024).

**Fig 9:**
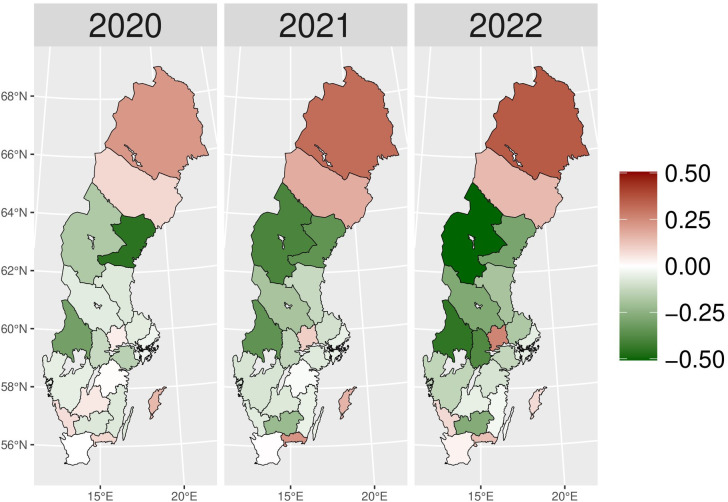
Regional differences in percentage changes of N07BC drug prescribing rates during the pandemic. Actual vs expected patients, both genders. Expected patient data based on the 2015–2019 average growth rate (source: Socialstyrelsen, 2024).

The prescribing of drugs used to treat opioid dependence (N07BC) shows an increasing trend throughout the pre-pandemic period (Graph 9). Starting from 2020, the actual values declined gradually until 2022. Regarding the regional variation in the prescribing of these drugs, Västerbottens, Norrbottens, and Jämtland counties in North Sweden showed a consistent increase over the pandemic period (Fig 9). Some counties in South Sweden, as well as Södermanland, also exhibited an increasing trend over time. Apart from these, the remaining counties experienced a decreasing rate of prescribing.

**Graph 10:**
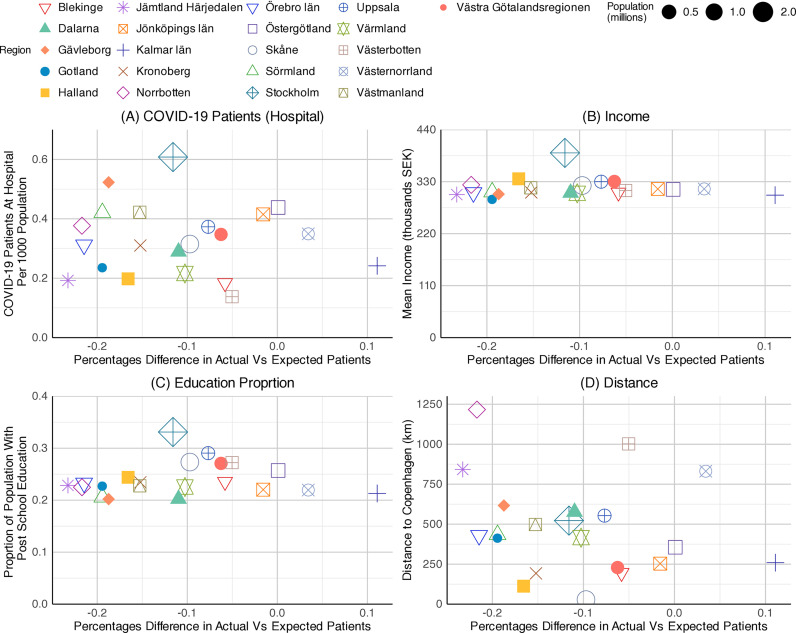
Scatterplots of N07BA (nicotine dependence drugs) patient differences by regions – the year 2020. Source: Socialstyrelsen (2024).

**Graph 11:**
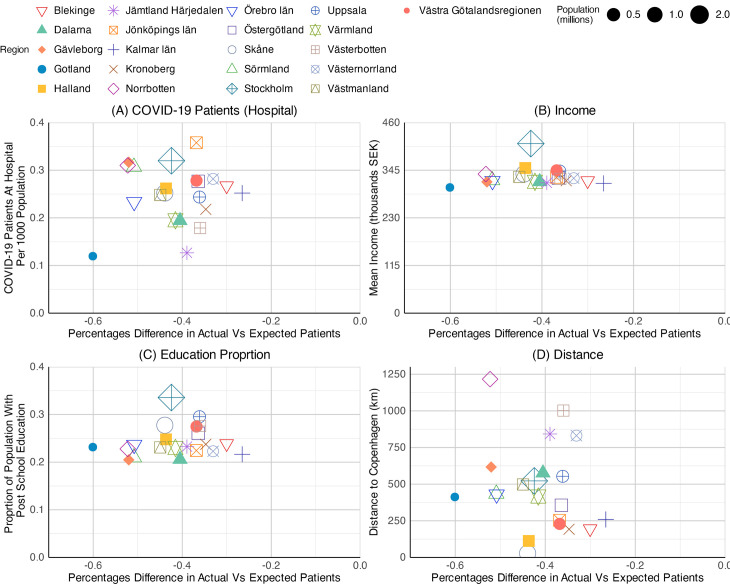
Scatterplots of N07BA (nicotine dependence drugs) patient differences by regions – the year 2021. Socialstyrelsen (2024).

**Graph 12:**
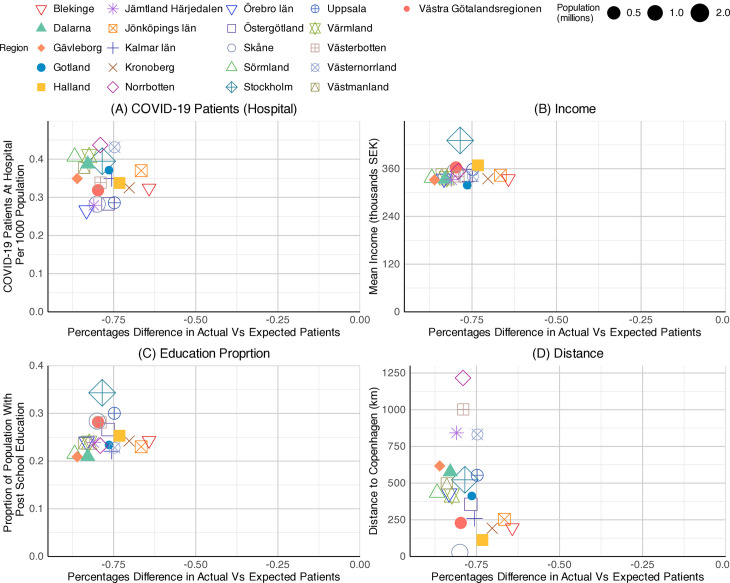
Scatterplots of N07BA (nicotine dependence drugs) patient differences by regions – the year 2022. Source: Socialstyrelsen (2024).

The percentage differences in actual vs. expected patients of N07BA (nicotine dependence drugs) were analysed against four chosen independent variables, including the COVID-19 patients at hospital per 1,000 population, the mean income, the proportion of the population with post-school education, and the distance from the capital to Copenhagen. Subsequently, these were presented as three sets of scatter plots, one for each year, 2020, 2021, and 2022 (Graphs 10–12).

The scatter plots for prescribing differences against COVID-19 inpatient rates showed a weak positive relationship in all three years. As described in the previous section, the gap in N07BA prescribing widened over the period, starting from 10 to −20 percent in 2020 (Graph 10), then from −20 to −70 percent in 2021 (Graph 11), and from −50 to −80 percent in 2022 (Graph 12). The COVID-19 patients at hospitals per 1,000 population were highest in Stockholm County at the start of the pandemic (Graph 10), with 0.6 patients per 1,000 population. The highest number declined in 2021, with 0.35 patients per 1,000 population in Jönköpings County (Graph 11). In 2022, Norrbottten County stood first with 0.45 patients per 1,000 population (Graph 12). However, the data points were too scattered to show a strong relationship between outcome and predictor variables. Among the regions, Stockholm County exhibited a moderate value, situated in the middle of the observed range, throughout the pandemic.

We found a weak association between mean income and the N07BA prescribing difference variables across all three years. The scatter plots for education proportion and prescribing differences showed a similar pattern to mean income. The correlation between the two variables remained insignificant; however, significant regional variations were seen throughout the pandemic. A slight negative pattern was observed in scatter plots of prescribing differences against distance to Copenhagen. The data points were relatively scattered in 2021, and a weak association was seen in 2020 and 2022. Nevertheless, the scatter plot showed no correlation between the response and explanatory variables.

Overall, none of the four variables showed a strong relationship with N07BA prescribing patterns across the observed years.

### Regression analysis

The whole pandemic period was analysed using the panel regression. In panel regression, the Hausman test was used to determine whether a fixed-effects or random-effects panel regression model was more appropriate for the analysis (S6 Table). As the Hausman test results appear statistically significant (χ² = 24.90, p < 0.001), the fixed-effects model was chosen for panel regression for the whole pandemic period of 2020 to 2022 (Table 1). Its results show a high R² value of 0.97 and an even higher adjusted R² value of 0.95, indicating that the model explains a large proportion of variance over time in the dependent variable (N07BA prescribing). Among the independent variables, only the mean income variable showed a statistically significant relationship with nicotine dependence drug prescribing, with a negative coefficient (β = -0.02, 95% CI: [-0.03, -0.01], p < 0.01).

**Table 1:** Fixed-effects panel regression results for nicotine dependence drug prescribing.

Variable	Fixed-effects Model (2020-2022)
COVID-19 patients at hospital per 1,000 population	−0.17 [-0.41, 0.07]
Mean income (thousand SEK)	−0.02 ^**^ [-0.03, -0.01]
Proportion of population with post-school education	−15.66 [-46.97, 15.64]
No. of Observations (N)	63
R^2^ (within)	0.97
Adj. R^2^	0.95

The dependent variable is the percentage difference in actual versus expected patients per 100,000 inhabitants for N07BA (Drugs used in nicotine dependence) prescribing. The outcome variable is in its original units. Values in brackets represent 95% confidence intervals.

** p < 0.01.

Additionally, the multiple linear regression models were used to perform regression analyses for the separate years 2020, 2021, and 2022 (S7 Table). All of these models show low R² values. No significant correlations were found between any of the investigated covariates and the differences in patients with N07BA in these regressions.

## Discussion

This study offers new insights into psychotropic medication prescribing patterns during the COVID-19 pandemic in Sweden, including regional (county-level) variations and the relationships between societal factors (which differ among Swedish counties) and psychopharmacological treatments throughout the pandemic years. We provide recommendations for public health policies to mitigate the long-term mental health impacts of a large-scale public health crisis. Our results also inform the health policies of other countries, providing more effective ways to prepare for and cope with public health demands during major emergencies. While some of the trends discovered were consistent with global patterns, others are unique to Sweden.

### Prescribing trends of psychotropic medicines in Sweden

The anxiolytic (N05B) prescribing in Sweden demonstrated a decreasing trend with a slight recovery in 2022. These findings align with the decrease in prescribing among newly diagnosed patients in 2020 in Germany [15]. It can be assumed that a less restrictive approach may reduce social restriction-related anxiety. Moreover, the Swedish society’s high trust in the government’s pandemic response [40] may also have contributed to the reduction of pandemic-induced anxiety and the need for anxiolytics. Another possible explanation is that the healthcare system in Sweden may have encouraged the use of non-pharmacological interventions for anxiety, such as stress management, rather than medication, while maintaining healthcare access.

The relatively stable prescribing patterns of hypnotic and sedative medications (N05C) observed in this study stand in contrast to the dynamic and shifting trends reported in international research findings. Globally, a significant increase in insomnia and sleep disturbances was observed [5,41]. In Denmark, the increased prescribing of benzodiazepine-related hypnotics was reported [21]. This stability pattern, followed by a slight decrease in most regions of Sweden throughout the pandemic, suggests that the Swedish population may have experienced a gradual recovery from insomnia. The possible explanation is that maintaining daily routines, facilitated by Sweden’s less restrictive approach to the pandemic, may have played a crucial role in preserving sleep patterns. This interpretation is supported by research highlighting the maintenance of sleep quality, especially during stressful events [42].

The observed regional variations in N05B and N05C prescribing also highlight the importance of considering local cultural factors in mental health interventions. For instance, less populated regions may have less pandemic-related anxiety and insomnia. The regionally differentiated coping mechanisms and unique local healthcare approaches may also play an important role.

The national data on antidepressant (N06A) prescribing shows a continuous increase in the years 2021 and 2022, which aligns with the global pattern of increasing depression during the COVID-19 pandemic [2,4,43]. The findings also correspond with the increased antidepressant prescribing patterns in the Scandinavian countries [21], the UK [22], Canada [24], and the new use of antidepressants during the pandemic in Australia [23]. Nevertheless, Sweden experienced a slight decrease at the start of the pandemic, which aligns with the study indicating no differences in depression symptoms during the first four weeks of the COVID-19 pandemic compared to the pre-pandemic period [3]. The Swedish population’s higher threshold for seeking medical intervention may have led to delayed anxiolytic prescribing in the early pandemic. While almost all counties experienced increased prescribing levels in 2022, regional variations persisted, particularly in 2021. The highest prescribing rates were observed in Västerbottens and regions in East Sweden, possibly due to variations in age distribution and population characteristics, such as the at-risk population and individual health-seeking behaviours. The local economic context could be another factor to consider because increased depression rates are highly associated with economic recessions and unemployment [44]. We took a closer look at the decreasing anxiolytic and increasing antidepressant prescribing. However, the number of medicines found within particular subgroups of N05B and N06C (the fifth level of the ATC classification) proved to be very small at the regional level, so even small changes, expressed in absolute measures, could have impacted the findings and subsequently influenced the interpretation of the results. An in-depth analysis of medicines prescribing at lower levels of ATC classification was beyond the scope of the current study.

The dramatic decrease in prescribing of all kinds of N07B drugs used in addictive disorders seems contrary to the international findings on increased addiction and use of addictive substances during the pandemic [20,45]. It is interesting to note that distinct regional variations were not seen in this drug group, except for Västernorrland and Kalmar counties (increased drug prescribing in 2020). Among the subgroups of N07B, the nicotine dependence drugs group (N07BA) showed the most striking reduction in prescribing. The smoking behaviours and cessation efforts during the COVID-19 pandemic reported varying results across the world, including both increased and decreased trends depending on the underlying reasons. The fear of COVID-19 led to a reduction in smoking, whereas mental health conditions like anxiety exacerbated the risky behaviour [46–50]. However, the current study findings align with the decrease in varenicline and nicotine replacement therapy (NRT) in Australia [51] and not meeting the expected prescription rates in the USA [52]. Possibly, the decreasing trends of the utilization of nicotine-dependence drugs observed in this analysis may be due to a shift towards non-prescription cessation therapy preference during the pandemic. Alternatively, it could indicate an increase in smoking during the pandemic or untreated nicotine dependence, both of which would have important public health implications. Yet another explanation could be the death of smokers during the COVID-19 pandemic.

The slight increases in the prescribing of medicines used in alcohol dependence treatment (N07BB), particularly in females, are consistent with the global trends of increased alcohol consumption during the pandemic [20,53]. This could indicate that some factors, such as gender-specific stressors (e.g., females are more stressed than males) [14], coping mechanisms applied during the COVID-19 pandemic, or shifting to home-based drinking culture (due to limitations in social gatherings) could have induced variations of N07BB prescribing between regions and genders.

Contrary to increased opioid use during the pandemic in some countries [20,45], a gradual decline in N07BC prescribing (drugs used in opioid dependence) has been observed in this analysis. It can be assumed that the Swedish healthcare system may have already implemented successful prevention and treatment in harm reduction programs. Hence, Sweden’s unique pandemic and healthcare approaches may offer essential insights into opioid management during crises.

In summary, apart from antidepressants, the prescribing of psychotropics showed a decreasing trend during the pandemic, particularly in the N07BA group of drugs used for nicotine dependence. The diverse trends across different drug classes and regional variations reflect the complexity of factors surrounding the psychotropic medication prescribing patterns in Sweden during the COVID-19 pandemic. Since healthcare accessibility in Sweden was maintained, these changes could be driven mainly by other factors, such as cultural, local, socio-economic, and demographic factors, rather than by barriers to accessing healthcare.

### Relationships between drug prescribing for nicotine dependence and socio-economic characteristics in Swedish regions

We found weak positive associations between the COVID-19 patients at hospital per 1,000 population and the NO7BA prescribing differences in the scatter plots for all three years. Some regions with higher COVID-19 patient numbers at hospitals tended to have N07BA prescribing patterns closer to expected values; however, the relationship was not statistically significant. The findings are consistent with reports from the UK, Italy, Spain, and Germany, where disease severity did not significantly impact the smoking cessation rate during the pandemic [54,55]. They also align with the decrease in attempts and success rate of quitting smoking during the pandemic, compared to the pre-pandemic period [54].

The analysis showed a weak positive relationship between the mean income and the N07BA prescribing differences in all observed years. Although some higher-income counties, such as Västra Götaland, showed less negative prescribing differences, the results varied across other counties and all three years. These weak relationships are contrary to the previously published studies, which reported higher smoking cessation success rates in individuals with higher education and full-time employment in Spain [54], and lower chances of lifestyle changes during the pandemic in unemployed groups in Sweden [9]. It is also essential to consider the social status and disparities of different Swedish community groups, which could be affected by Sweden’s unique pandemic approach [56].

Although some counties with higher post-school education levels, such as Uppsala and Västra Götaland showed a more positive prescribing pattern, the overall correlation is not significant. This partially aligns with the reports from other studies on the increased psychological distress in lower education level groups [43] and a higher rate of quitting smoking in individuals with higher education backgrounds during the pandemic [54]. The relationships between the distance from the capital to Copenhagen and N07BA prescribing remained weak throughout the pandemic years. There may be an impact of travel restrictions on access to cross-border purchasing of nicotine products and alcohol, as Sweden has higher taxes on these products than Denmark or Germany [35,57].

None of the variables showed a significant correlation with the N07BA drug prescribing in the initial testing with scatter plots. A persistent weak relationship and extensive regional variations in all variables across all three years highlight the complex nature of factors affecting the changes in medication prescribing patterns. Other factors, such as regional policies, healthcare literacy, and localised socio-economic factors in different community groups, may also have combined effects on prescribing trends throughout the pandemic.

### Interpreting the relationships tested in the regression analysis

A fixed-effects panel regression over the entire period (2020–2022) was employed. As far as the education variable was concerned, the regression model showed an insignificant correlation. Some other studies reported that lower education levels are associated with higher levels of mental health problems [2,43]. In contrast, one study in China found the opposite finding that individuals with higher education are more prone to depression during the pandemic [58]. These inconsistent results highlight the complex role of education levels in health behaviour during crises. In addition to education levels, there could be several mediating factors to the changes in nicotine dependence drug prescribing, which are not captured in the current model.

In our fixed-effects panel regression for 2020–2022, the mean income showed a significant negative correlation. It appears that the income factor is correlated with the temporal variation between regions during that period. Although the results align with the published reports [2,43], mentioning the association between lower socio-economic status and higher risk of mental health symptoms, the mean income variable in this study could not explain the continuous decline in nicotine dependence drug prescribing throughout the pandemic. Potentially, the statistically significant correlation between the mean income and nicotine dependence drug prescribing could be related to several socio-economic mechanisms, including, e.g., reduced access to non-pharmacological interventions among less affluent patients and their financial instability.

Additionally, linear regression was applied for each of the individual years (2020, 2021, 2022). These analyses found no statistically significant relationship between the COVID-19 patients at the hospital and N07BA prescribing patterns. Surprisingly, the tests of the possibility of influence on N07BA prescribing by the disruption in cross-border purchasing factor (using the distance to Copenhagen variable as a covariate in multiple regression analysis for each year) did not show any significant correlation either.

None of these chosen variables could fully explain the decline of N07BA drugs. This also suggests that other variables, not measured in this study, should be considered in future research. Nevertheless, a temporal effect is present throughout the entire period, which is strongly correlated with the income factor.

### Public policy implications and recommendations

Most of the psychotropic medications, except antidepressants and hypnotics/sedatives, experienced decreasing prescribing trends during the pandemic, which was the most prominent in drugs used in nicotine dependence (N07BA). None of the studied factors could explain the initial reduced levels in 2020. However, socioeconomic factors, particularly income-related factors, play a significant role in changes in N07BA prescribing during the pandemic period. The negative correlations in the panel regression highlight that health authorities should consider increasing targeted mental health and smoking cessation awareness campaigns tailored to educational backgrounds in regions with higher income. Although the coefficient for the education factor is insignificant, the positive effect highlights that education influences the long-term management of nicotine dependence rather than abrupt changes in prescribing behavior during the pandemic. Implementing economic support could help to reduce the income-related disparities during the pandemic, as well as indirectly support coping with mental health problems arising from socio-economic inequalities. Moreover, policymakers should emphasize developing region-specific mental health or addiction policies, providing autonomy to regional health authorities, and adapting to local circumstances, especially in times of crisis.

The lack of clear correlations between COVID-19 hospitalization rates and N07BA prescribing levels suggests that the disease spread factor also could not explain the changes in drug prescribing trends. Nevertheless, health authorities should incorporate mental health and addiction treatment strategies into health plans as well as develop a flexible regulatory framework for the adaptation of mental health services during emergencies. In the event of future health crises, integrated public health strategies should be developed in conjunction with emergency health mitigation interventions. A real-time and long-term monitoring system implemented during and after the pandemic or other public health emergencies could support addressing mental healthcare needs.

### Limitations and future research directions

The study has several limitations. First, the data available in this study were limited and subject to external generalizability constraints. Second, the data exclude OTC and hospital prescribing; therefore, to mitigate potential bias, the study results, including those on the use of N07BA drugs, were interpreted carefully. The lack of including OTC medicines in this study may affect the generalizability of the findings, particularly for vulnerable populations or other patient groups that do not regularly seek and/or obtain professional medical services. Third, the study results reflect the Swedish health system, which may not be generalizable to other countries with their distinct health delivery systems, comprising unique legislative and organizational characteristics, and within diverse clinical and political settings.

Future research, including OTC and non-pharmacological interventions, is recommended to characterize prescribing trends across Sweden. Subsequent studies should investigate other related and additional socioeconomic factors that may influence changes in medication prescribing during health emergencies. A further longitudinal study on the long-term relationships between income and education factors and psychotropic medication prescribing patterns would be helpful. To understand the localized factors and disparities, a detailed analysis at the municipality level could also be performed. Moreover, qualitative studies, including interviews and focus groups, would provide deeper insights into exploring the causal factors. Subgroup analyses are also recommended to identify prescribing patterns in specific groups, such as those based on age, gender, and ethnicity. An in-depth analysis of decreasing anxiolytic and increasing antidepressant prescribing, going down to the lowest levels of the ATC classification, could help to explain both of these intriguing phenomena.

## Conclusions

The prescribing patterns of anxiolytics and other drugs used for addictive disorders during the COVID-19 pandemic in Sweden experienced distinct decreasing trends. In contrast, antidepressants experienced a slight increase over the period, and hypnotics and sedatives showed a relatively stable trend. The most striking reductions were seen in drugs used to treat nicotine dependence (N07BA), and significant regional variations were observed across Sweden. In the regression analysis, none of the key variables showed a statistically significant correlation with the changes in N07BA drug prescribing. The negatively significant income factor in the panel regression during the pandemic period indicates the presence of a temporal effect that strongly correlates with this factor. However, the pandemic effects differed across the regions, which the four explanatory variables could not adequately explain. Policymakers should emphasize targeted mental health interventions to alleviate the socio-economic disparities and consider developing region-specific mental health and addiction policies for addressing regional variations during health emergencies.

## Supporting information

S1 Data Population as of 1 November, number by age, sex, year, and region. (XLSX)

S2 Table The detailed description of the geographical administrative division of Sweden. (DOCX)

S3 Text The detailed information on psychotropic medication data extraction and processing. (DOCX)

S4 Text The socio-economic data measure. (DOCX)

S5 Data Prescription data. (XLSX)

S6 Table The Hausman test results. (DOCX)

S7 Table Multiple linear regression results for nicotine dependence drug prescribing for 2020, 2021, and 2022. (DOCX)
