# Supplementary material for: Correlations between the prescribing patterns of psychotropic medications and socio-economic factors during the COVID-19 pandemic: A cross-sectional Swedish registry study
Source: PLoS One. 2025 Sep 17;20(9):e0330081. doi: 10.1371/journal.pone.0330081 (PMC12443284; doi:10.1371/journal.pone.0330081)
Supplement: S2 Table — (DOCX) [file pone.0330081.s002.docx]

S2 Table. The detailed description of the geographical administrative division of Sweden.

Table of Administrative Regions of Sweden (Source: The Government Offices of Sweden, 2015)

| EAST SWEDEN | SOUTH SWEDEN | NORTH SWEDEN |
| --- | --- | --- |
| East Middle Sweden  Södermanlands län  Östergötlands län  Örebro län  Västmanlands län  Uppsala län | **Småland and the islands**  Jönköpings län  Kronobergs län  Kalmar län  Gotlands län | **North Middle Sweden**  Värmlands län  Dalarnas län  Gävleborgs län |
| Stockholm  Stockholms län | **South Sweden**  Blekinge län  Skåne län | **Middle Norrland**  Västernorrlands län  Jämtlands län |
|  | **West Sweden**  Hallands län  Västra Götalands län | **Upper Norrland**  Västerbottens län  Norrbottens län |
