# Supplementary material for: Correlations between the prescribing patterns of psychotropic medications and socio-economic factors during the COVID-19 pandemic: A cross-sectional Swedish registry study
Source: PLoS One. 2025 Sep 17;20(9):e0330081. doi: 10.1371/journal.pone.0330081 (PMC12443284; doi:10.1371/journal.pone.0330081)
Supplement: S3 Text — (DOCX) [file pone.0330081.s003.docx]

S3 Text. The detailed information on psychotropic medication data extraction and processing.

**Data Extraction:**

The interactive web-based database contains comprehensive information on all dispensed prescription drugs in Sweden, including the patient's age, gender, region, and the prescribed drug's ATC classification code. The Swedish Pharmaceutical database has a high level of completeness and accuracy, as it covers all dispensed prescription drugs at pharmacies in Sweden. These four drug classes with subclasses were selected based on the literature reviews, indicating the high prevalence of COVID-19 pandemic-associated mental health symptoms. The period was selected from 2014 to 2022 for a longitudinal analysis of drug prescription patterns over time. After selecting the required ATC code and parameters of interest, the data was downloaded in JSON format. The data are downloaded with stratification by sex (Male, Female, Male, and Female), age class (0-4,10-14,15-29, 30-39, 40-49, 50-59, 60-69, 70-79, 80+), and region (21 counties and the whole Sweden).

**Data processing:**

After data extraction, the JSON files were processed and combined into a single data frame, and then database codes were formatted into meaningful labels. Initial data processing steps, including data download and formatting, were performed using R code provided by IHE-Sweden (the Institute of Health Economics) as part of a collaborative project. Since the data obtained from the Socialstyrelsen API was already structured, no specific data-cleaning techniques were required. However, data processing was done to facilitate further analysis. The drug prescription data was processed and analyzed using R version 4.4.0 and RStudio version 2024.04.1.
