# Supplementary material for: Correlations between the prescribing patterns of psychotropic medications and socio-economic factors during the COVID-19 pandemic: A cross-sectional Swedish registry study
Source: PLoS One. 2025 Sep 17;20(9):e0330081. doi: 10.1371/journal.pone.0330081 (PMC12443284; doi:10.1371/journal.pone.0330081)
Supplement: S4 Text — (DOCX) [file pone.0330081.s004.docx]

S4 Text. The socio-economic data measure.

**Socio-economic (Independent variables) data:**

The income data from the Household Finances database of Statistics Sweden (Statistiska centralbyrån, SCB) is available in Swedish Krona (SEK) in thousands, and the data exclude the information of individuals without disposable family income and household information that is unavailable.

The education database is classified according to the Swedish National Educational Classification (SUN) and there are 7 classes in the database. Two education classes: post-secondary education of less than 3 years (ISCED97 4+5B) and 3 years or more (ISCED97 5A) were selected for the ‘proportion of population with post-school education’ variable for the analysis.

In Sweden, alcohol sale is strictly controlled by the Systembolaget of the Swedish Government, which has a monopoly on retail sales of alcohol [1]. Therefore, the individual use and restaurant use of alcohol sales are regulated as well, and the taxes are higher compared to Denmark (reduction in tax by 45% in 2003) [2]. It can be hypothesized that travel restrictions could change alcohol and nicotine consumption during the pandemic as people from Sweden tended to buy alcohol and nicotine products from neighbouring countries before COVID-19. Therefore, to explore whether this cross-border purchasing was affected by travel restrictions during the pandemic and whether this impact could in turn affect the reduction in N07BA drug prescribing, the distance to Copenhagen (km) variable was chosen for regression analysis.

Sources:

1. Systembolaget. Systembolaget explained [Internet]. 2024 [cited 2024 Sep 12]. Available from: <https://www.omsystembolaget.se/english/systembolaget-explained/>
2. Gustafsson NK. Changes in Alcohol Availability, Price and Alcohol-related Problems and the Collectivity of Drinking Cultures: What Happened in Southern and Northern Sweden? Alcohol Alcohol. 2010;45(5):456–67.
