# Supplementary material for: Correlations between the prescribing patterns of psychotropic medications and socio-economic factors during the COVID-19 pandemic: A cross-sectional Swedish registry study
Source: PLoS One. 2025 Sep 17;20(9):e0330081. doi: 10.1371/journal.pone.0330081 (PMC12443284; doi:10.1371/journal.pone.0330081)
Supplement: S6 Table — (DOCX) [file pone.0330081.s006.docx]

**S6 Table.** **The Hausman test results.**

| Model = y ~ x1 + x2 + x3 + x4 | | | |
| --- | --- | --- | --- |
| **Hausman Test** | Chi-square Statistic | Degrees of Freedom | p-value |
|  | 24.90 | 3 | 0.00001623 |
| alternative hypothesis: one model is inconsistent | | | |
