# Supplementary material for: Correlations between the prescribing patterns of psychotropic medications and socio-economic factors during the COVID-19 pandemic: A cross-sectional Swedish registry study
Source: PLoS One. 2025 Sep 17;20(9):e0330081. doi: 10.1371/journal.pone.0330081 (PMC12443284; doi:10.1371/journal.pone.0330081)
Supplement: S7 Table — (DOCX) [file pone.0330081.s007.docx]

**S7 Table. Multiple linear regression results for nicotine dependence drug prescribing for 2020, 2021, and 2022.**

| **Variable** | **Model 1**  **(2020)** | **Model 2**  **(2021)** | **Model 3**  **(2022)** |
| --- | --- | --- | --- |
| (Intercept) | -0.02  [-0.96, 0.93] | -0.21  [-0.99, 0.57] | -0.71 *  [-1.20, -0.22] |
| COVID-19 patients at hospital per 1,000 population | -0.02  [-0.51, 0.47] | 0.32  [-0.59, 1.23] | 0.21  [-0.60, 1.02] |
| Mean income | -0.00  [-0.01, 0.00] | -0.00  [-0.01, 0.00] | -0.00  [-0.00, 0.00] |
| Proportion of population with post-school education | 0.62  [-1.89, 3.13] | 1.27  [-1.21, 3.75] | 0.73  [-1.18, 2.63] |
| Distance to Copenhagen (km) | -0.00  [-0.00, 0.00] | -0.00  [-0.00, 0.00] | -0.00  [-0.00, 0.00] |
| No. of Observations (N) | 21 | 21 | 21 |
| R^2^ (within) | 0.07 | 0.11 | 0.14 |
| Adj. R^2^ | -0.16 | -0.12 | -0.07 |

The dependent variable is the percentage difference in actual vs expected patients per 100,000 inhabitants for N07BA (Drugs used in nicotine dependence) prescribing. All continuous predictors are mean-centered and scaled by 1 standard deviation. The outcome variable is in its original units. Confidence intervals in brackets. * p < 0.05
